# A Metric Learning-Based Improved Oriented R-CNN for Wildfire Detection in Power Transmission Corridors

**DOI:** 10.3390/s25133882

**Published:** 2025-06-22

**Authors:** Xiaole Wang, Bo Wang, Peng Luo, Leixiong Wang, Yurou Wu

**Affiliations:** 1 School of Electrical Engineering and Automation, Wuhan University, Wuhan 430072, China; 2024202070023@whu.edu.cn (X.W.); thornluo@whu.edu.cn (P.L.); 2 Wuhan Power Supply Company, State Grid Hubei Electric Power Company, Wuhan 430013, China; leixiong2024@foxmail.com (L.W.); yurou1028@foxmail.com (Y.W.)

**Keywords:** wildfire detection, transmission corridors, metric learning, group convolutions, content-aware up-sampling

## Abstract

Wildfire detection in power transmission corridors is essential for providing timely warnings and ensuring the safe and stable operation of power lines. However, this task faces significant challenges due to the large number of smoke-like samples in the background, the complex and diverse target morphologies, and the difficulty of detecting small-scale smoke and flame objects. To address these issues, this paper proposed an improved Oriented R-CNN model enhanced with metric learning for wildfire detection in power transmission corridors. Specifically, a multi-center metric loss (MCM-Loss) module based on metric learning was introduced to enhance the model’s ability to differentiate features of similar targets, thereby improving the recognition accuracy in the presence of interference. Experimental results showed that the introduction of the MCM-Loss module increased the average precision (AP) for smoke targets by 2.7%. In addition, the group convolution-based network ResNeXt was adopted to replace the original backbone network ResNet, broadening the channel dimensions of the feature extraction network and enhancing the model’s capability to detect flame and smoke targets with diverse morphologies. This substitution led to a 0.6% improvement in mean average precision (mAP). Furthermore, an FPN-CARAFE module was designed by incorporating the content-aware up-sampling operator CARAFE, which improved multi-scale feature representation and significantly boosted performance in detecting small targets. In particular, the proposed FPN-CARAFE module improved the AP for fire targets by 8.1%. Experimental results demonstrated that the proposed model achieved superior performance in wildfire detection within power transmission corridors, achieving a mAP of 90.4% on the test dataset—an improvement of 6.4% over the baseline model. Compared with other commonly used object detection algorithms, the model developed in this study exhibited improved detection performance on the test dataset, offering research support for wildfire monitoring in power transmission corridors.

## 1. Introduction

The safe and stable operation of the power system is fundamental to the development of the national economy. In recent years, due to increasingly dry climatic conditions and frequent wildfires, numerous transmission lines have been disrupted by flame and smoke, leading to aerial power line faults and posing serious threats to grid safety and stability [1,2,3]. Consequently, effective monitoring and prevention strategies are urgently needed to mitigate the adverse impacts of wildfires on the power industry [4,5,6]. Traditional monitoring approaches typically rely on manual inspections of wildfire-prone areas along transmission corridors; however, such methods are inefficient, labor-intensive, and incapable of providing continuous coverage across wide regions, often resulting in delayed responses and increased risk of wildfire-related incidents [7]. Therefore, developing real-time, online monitoring techniques for wildfire detection in transmission corridors is of great significance for safeguarding the secure and stable operation of the power grid.

Currently, the primary online monitoring methods for wildfires in power grids include satellite remote sensing [8,9], LiDAR scanning [10,11,12], and image/video-based surveillance [13]. Among them, satellite remote sensing is often susceptible to interference from clouds and non-wildfire thermal sources [14], while LiDAR systems can be significantly affected by adverse weather conditions such as heavy fog. These limitations hinder their reliability and applicability in real-time wildfire monitoring scenarios.

With the ongoing digitalization of power systems, the deployment of image and video surveillance equipment along transmission lines has become increasingly widespread. Wildfire detection technologies based on image and video data offer advantages, such as rapid response and low cost, making them highly attractive for practical applications. Wildfire image and video detection typically consists of two main tasks: flame detection and smoke detection.

In recent years, deep learning-based image and video monitoring techniques have advanced rapidly. According to their detection paradigms, these methods are broadly categorized into single-stage and two-stage detection algorithms. Single-stage algorithms, such as YOLO and SSD, are known for their high detection speed. For instance, reference [15] proposed a lightweight YOLOv5 variant by simplifying the neck network structure to enable real-time wildfire monitoring in power transmission scenarios. However, single-stage methods often suffer from relatively lower detection accuracy.

In contrast, two-stage detection algorithms typically operate in two sequential steps: the first stage generates a set of candidate object proposals based on extracted features, while the second stage performs fine-grained classification and bounding box regression [16]. Although less efficient, these methods generally achieve higher accuracy and better generalization, making them more suitable for tasks like flame and smoke detection that require precise recognition.

Reference [17] embedded the weakly supervised learning strategy into the Faster-RCNN framework to improve the model’s ability to extract features of flames and smoke. Reference [18] introduced a parallel attention module (PAM) into the region proposal network (RPN), where the combined use of channel and spatial attention mechanisms significantly improved flame recognition accuracy. Moreover, Oriented R-CNN, as proposed in [19], utilizes rotated bounding boxes to better capture irregular objects with arbitrary orientations and shapes, making it well-suited for detecting smoke and flame patterns distorted by wind or other environmental factors. However, when applied directly, its recognition accuracy remains suboptimal, especially under complex background interference and feature ambiguity. In power transmission corridor scenarios, existing Oriented R-CNN models are prone to misidentifying smoke targets due to visually similar background interference and often fail to accurately detect small-scale wildfire instances.

In the task of wildfire detection within power transmission corridors, smoke targets often exhibit high visual similarity to background elements, such as clouds, water reflections, and distant blurred terrain. Since computer vision algorithms primarily rely on low-level visual cues—such as local texture, color, and transparency—while lacking high-level semantic understanding, they are particularly prone to feature confusion and misclassification when encountering smoke-like backgrounds [20,21,22]. Moreover, both smoke and flame targets exhibit highly variable and dynamic shapes throughout the combustion process, which further blurs their visual features and complicates the extraction of stable and discriminative representations. Additionally, the spatial scale of wildfire targets varies significantly [23,24]; in the early stages of a wildfire, both smoke and flame regions tend to occupy only small portions of the image, making feature extraction especially challenging. These factors collectively exacerbate the difficulty of accurate and robust wildfire detection in power transmission environments.

To address the above challenges, this paper proposed a wildfire detection method for power transmission corridors based on an improved Oriented R-CNN model enhanced with metric learning. The proposed approach integrated three key modules: a multi-feature center metric loss (MCM-Loss) module derived from metric learning principles, the group convolutional backbone ResNeXt [25], and the content-aware reassembly of features (CARAFE) up-sampling operator [26].

Taking the Oriented R-CNN model as the baseline, the major contributions and innovations of this paper are summarized as follows:

(i) A multi-feature center metric loss (MCM-Loss) module based on metric learning was proposed to enhance the model’s ability to distinguish between smoke and visually similar background samples. This effectively reduced false positives and missed detections and improved the overall recognition accuracy for smoke targets.
(ii) The original ResNet backbone in the Oriented R-CNN was replaced with the group convolution network ResNeXt, which expanded the channel capacity for feature extraction without increasing model complexity, thereby enhancing the model’s performance in detecting flames and smoke with complex shapes and varying morphologies.
(iii) An FPN-CARAFE structure was proposed by integrating the content-aware up-sampling operator CARAFE into the traditional feature pyramid network (FPN), which improved multi-scale feature fusion and preserved fine-grained information, leading to more accurate detection of small and irregular wildfire targets.

Together, these components enhanced the network’s ability to extract and represent discriminative features from complex wildfire imagery, thereby improving detection accuracy and robustness. The proposed method was trained and evaluated on a dedicated dataset of wildfire images in power transmission scenarios. Experimental results demonstrated that the model achieved superior performance in terms of accuracy, recall, and average precision in both flame and smoke detection tasks, compared to baseline and other commonly used detection algorithms.

## 2. The Metric Learning-Based Wildfire Detection Model for Power Transmission Corridors

### 2.1. A Framework for Wildfire Detection Based on Metric Learning

**Figure 1:**
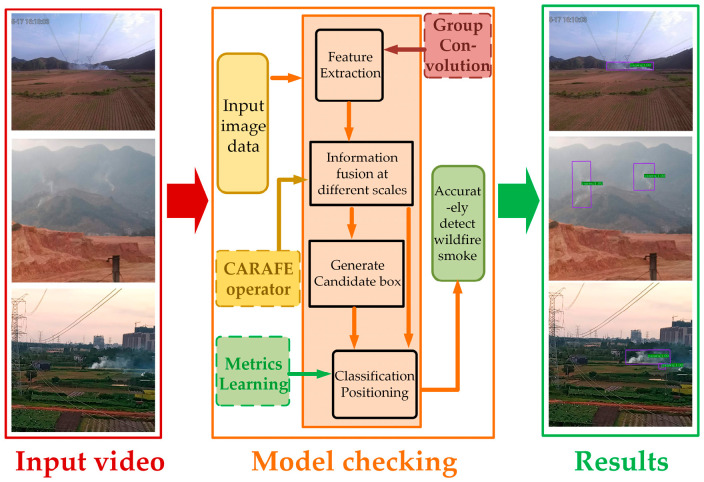
Overall detection framework.

In wildfire detection tasks, flames and smoke often spread in different directions and at varying angles, making it difficult for horizontal bounding boxes to accurately localize targets. To address this issue, this paper adopted the Oriented R-CNN, a rotated bounding box detection algorithm, as the baseline model due to its suitability for capturing arbitrarily oriented targets. Building upon this framework, an improved Oriented R-CNN model incorporating metric learning was proposed for wildfire detection in power transmission corridors. The overall detection framework is illustrated in Figure 1.

### 2.2. Overview of the Oriented R-CNN Model

**Figure 2:**
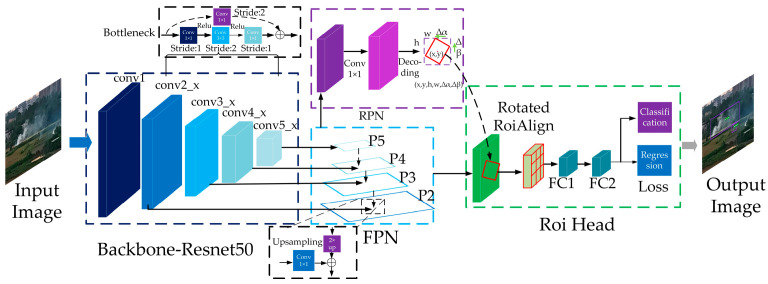
The network architecture of the Oriented R-CNN model.

Oriented R-CNN is a two-stage rotated object detection model based on region proposals. Compared with other commonly used rotated bounding box detection algorithms, it adopts a midpoint offset representation to generate oriented proposals, which reduces computational redundancy and improves overall efficiency. The model primarily consists of five components: the input module, the backbone network, the FPN, the oriented region proposal network (oriented RPN), and the detection head. The overall network architecture is illustrated in Figure 2.

First, the images collected around the transmission lines are preprocessed and passed through the backbone network ResNet for feature extraction via convolutional operations. The extracted features are then fused through the FPN layer to generate multi-scale feature maps of flame and smoke targets. Subsequently, the up-sampled deep features are fused with the shallow, fine-grained features, integrating detailed information across scales. These fused features are fed into the oriented RPN, a lightweight, fully convolutional network with six regression parameters, to generate rotated proposal boxes. Meanwhile, the features are also passed to the detection head, which performs classification and refines the spatial positions of the proposals. Ultimately, the model outputs detection results that include the category, location, and confidence score of the flame and smoke targets.

Compared with traditional detection methods, Oriented R-CNN employs rotated bounding boxes, making it more suitable for detecting irregular flame and smoke regions in the wild, which are often influenced by wind and other environmental factors. However, the task of wildfire detection remains challenging. Enhancing the accuracy of wildfire recognition by improving the Oriented R-CNN model is therefore the primary objective of this study.

### 2.3. Multi-Feature Center Metric Loss (MCM-Loss) Module Based on Metric Learning

**Figure 3:**
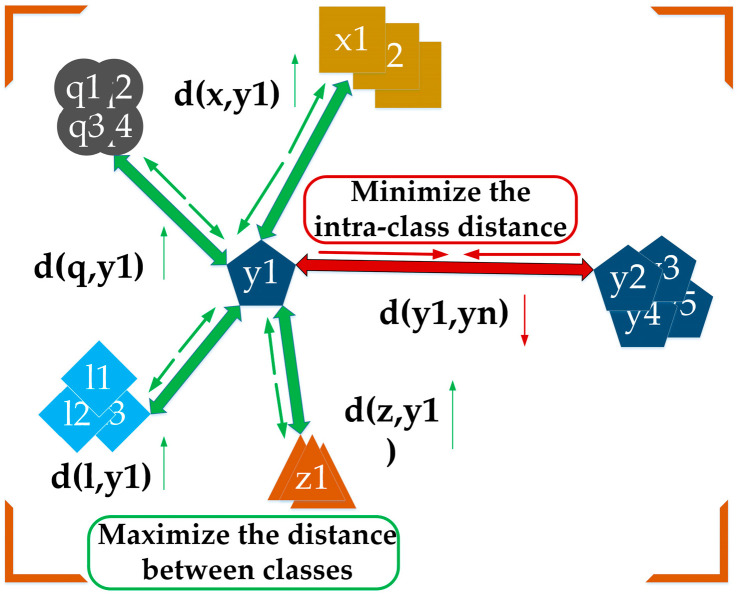
Principles of metric learning.

Metric learning is a method based on distance measurement, designed to capture the similarities or differences between samples. It aims to reduce the distance between samples of the same category while increasing the distance between samples from different categories [27,28,29]. The principle of metric learning is illustrated in Figure 3.

Wildfire images are frequently affected by severe background interference, particularly because the smoke category contains a large number of visually similar samples. Inspired by the principle of metric learning—which aims to maximize inter-class distances while minimizing intra-class distances to effectively distinguish between easily confused target categories—this paper designed a multi-feature center metric loss (MCM-Loss) module based on metric learning.

Traditional metric learning methods typically rely on a single feature center for each category [30]. However, the feature distribution of real-world smoke data is often complex and highly variable. Smoke targets exhibit strong nonlinearity and dynamic characteristics—their diffusion is influenced by factors such as wind direction, temperature, and humidity, leading to diverse distribution patterns across different temporal and spatial scales. To address this, the MCM-Loss module proposed in this paper assigned multiple initialized feature centers to each category, enabling a more accurate representation of intra-class feature variations and improving the model’s adaptability to smoke detection tasks involving highly variable target morphologies.

Specifically, the initial prototype feature center library is defined as:

(1) $$P={\left\{p_{c,j}\right\}}_{c=1,j=1}^{C+1,K}$$

Among them, P is the set of proxy feature centers of all categories; $p_{c,j}$ is the jth feature center of the c category; C is the total number of categories (class-num); and K is the number of feature centers of each category (num-proxies), which was set to 3 in the experiments in this paper.

These feature centers are defined as trainable parameters:

(2) $$P\in {\mathbb{R}}^{(C+1)\times K\times d}$$

Among them, d is the feature dimension, and the additional (C + 1)th category is used for the background class feature center.

At the beginning of training, the feature centers were initialized with random values sampled from a Gaussian distribution and optimized using the Kaiming normal initialization method, ensuring a well-distributed starting point that facilitated effective convergence in the early stages of training.

To dynamically update the feature centers and better adapt to the evolving sample distribution, this paper adopted the exponential moving average (EMA) strategy for center updates:

(3) $$p_{c,j}^{\left(t+1\right)}=\frac{0.9p_{c,j}^{\left(t\right)}+0.1\bar{x_{c}}}{\left\|0.9p_{c,j}^{\left(t\right)}+0.1\bar{x_{c}}\right\|}$$

The mean feature $\bar{x_{c}}$ of the sample of the c category is defined as shown in Equation (4):

(4) $$\bar{x_{c}}=\frac{1}{N_{c}}\sum_{i\in S_{c}}x_{i}$$

$S_{c}$ represents the set of all samples of category c in a batch. This updating method makes the feature center gradually approach the actual data distribution and will not be affected by the drastic fluctuations of a single batch of data.

The basis of metric learning is to represent the distance between two samples. This paper used cosine similarity as the distance measurement method between the sample and feature center, as shown in Equation (5):

(5) $$s_{i,j}=\cos(x_{i},p_{i})=\frac{x_{i}\cdot p_{i}}{\left\|x_{i}\right\|\left\|p_{i}\right\|}$$

where $s_{i,j}$ represents the cosine similarity between the two, with a value range of [−1, 1].

Introducing metric learning requires determining positive and negative samples. For a sample $x_{i}$ (y category), all feature centers of the y category belonged to positive samples $P_{i}$, while feature centers of all other categories belonged to negative samples $N_{i}$:

(6) $$P_{i}={\left\{p_{y,j}\right\}}_{j=1}^{K}$$

(7) $$N_{i}={\left\{p_{z,j}\right\}}_{z\neq y,j=1}^{K}$$

When calculating the metric learning loss function, the positive sample loss $L_{pos}$ was weighted by the similarity between the sample $x_{i}$ and all positive sample feature centers, and the negative sample loss $L_{neg}$ was weighted by the similarity between the sample $x_{i}$ and all negative sample feature centers:

(8) $$L_{pos}=\frac{1}{N}\sum_{i}\log(1+\sum_{j=1}^{K}e^{-\alpha (s_{i,y,j}-m)})$$

(9) $$L_{neg}=\frac{1}{N}\sum_{i}\log(1+\sum_{z\neq y,j=1}^{K}e^{\alpha (s_{i,z,j}+m)})$$

Among them, α controls the loss magnification, and m is the boundary hyperparameter (margin), which is used to increase the discrimination. In our implementation, the scaling factor α was set to 32, and the margin m was set to 0, based on empirical tuning on the validation set.

To enhance the model’s discriminative capability, we assigned greater weight to additional hard positive and hard negative samples during training. This strategy was motivated by the fact that these difficult samples were more prone to misclassification and thus required greater attention during learning. By explicitly focusing on these samples, the model can better learn to distinguish fine-grained differences.

For a given sample $x_{i}$, the hard positive was defined as the positive sample with the lowest cosine similarity to it, while the hard negative was the negative sample with the highest cosine similarity:

(10) $$s_{i,y}^{\min}=\underset{j}{\min}s_{i,y,j}$$

(11) $$s_{i,z}^{\max}=\underset{z\neq y,j}{\max}s_{i,z,j}$$

For hard positive and hard negative samples, the metric learning loss values were calculated separately. The computation followed the same principles as described in Equations (8) and (9).

(12) $$L_{hard-pos}=\frac{1}{N}\sum_{i}\log(1+e^{-\alpha (s_{i,y}^{\min}-m)})$$

(13) $$L_{hard-neg}=\frac{1}{N}\sum_{i}\log(1+e^{\alpha (s_{i,z}^{\max}+m)})$$

The final loss was composed of four components: the positive sample loss, the negative sample loss, the hard positive sample loss, and the hard negative sample loss. The weight coefficients for the hard positive and hard negative sample losses were both set to 2, with the goal of enhancing the model’s focus on difficult samples and guiding the network to pay more attention to those that are challenging to distinguish:

(14) $$L=(L_{pos}+L_{neg})+2(L_{hard-pos}+L_{hard-neg})$$

**Figure 4:**
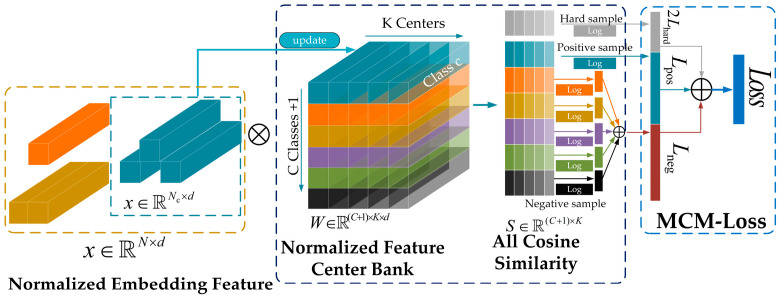
Working principle of the multi-feature center metric loss (MCM-Loss) module.

Based on this, the working principle of the multi-feature center metric loss (MCM-Loss) module based on metric learning is illustrated in Figure 4.

As shown in the figure, the multi-feature center metric loss (MCM-Loss) module updated the model’s weight parameters through the metric learning loss function, enhancing the model’s ability to distinguish between visually similar samples. This improved the recognition accuracy for easily confused targets, thereby enhancing overall detection performance in the wildfire detection task for power transmission corridors.

### 2.4. Group Convolutional Structure ResNeXt Replaces the Backbone Network

When processing flame and smoke targets, traditional convolution methods often result in the mixing of extracted information, such as color and texture, due to the lack of specificity among different feature channels during the feature extraction process. Moreover, flames typically exhibit localized high-brightness regions, while smoke is characterized by large-scale diffusion. A single feature extraction and fusion approach struggles to capture these distinct scales and forms simultaneously, thereby compromising detection performance.

**Figure 5:**
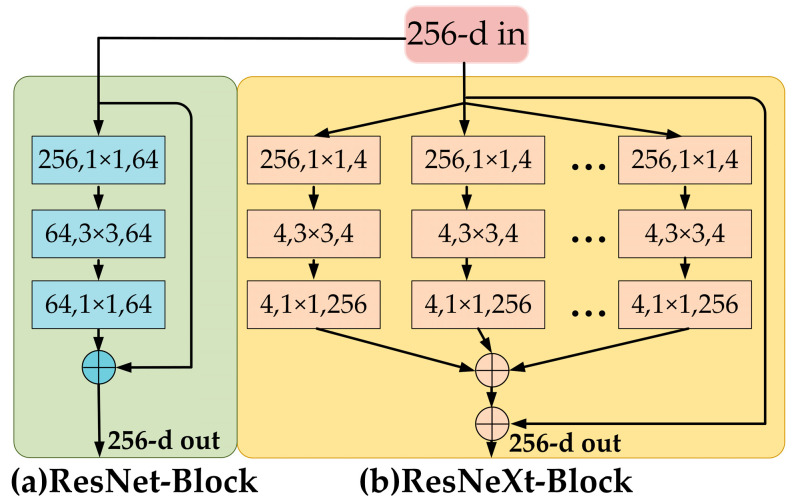
Comparison of the block structures of ResNet and ResNeXt.

To expand the channel domain of the feature extraction network without increasing the number of model parameters and to enhance the feature extraction capability for detecting flames and smoke in power transmission lines, this paper adopted the ResNeXt network, which is based on the concept of group convolution. ResNeXt is a significant improvement over the ResNet architecture. While retaining the advantages of ResNet’s residual connections, it successfully incorporates the multi-branch design concept from the Inception series [31], introducing a more innovative grouping strategy that offers strong architectural flexibility and superior performance. The block structure comparison between ResNet and ResNeXt is illustrated in Figure 5.

From an overall perspective, the basic unit (block) of ResNeXt is similar to that of ResNet, retaining the shortcut residual connection to alleviate the gradient vanishing problem commonly encountered in deep networks. However, by widening and grouping the network, ResNeXt achieves more efficient feature representation capabilities. In practical applications, the topological structure within each group is typically consistent, which makes the network more modular and reusable. This not only simplifies model construction but also significantly reduces the effort required for hyperparameter tuning. Group convolution divides the channels into multiple subgroups, with each group performing convolution operations independently. This effectively achieves feature decoupling and parallel learning. Specifically, this mechanism enables the network to learn features across different dimensions—such as color, texture, and shape—in various channel domains and then fuse the outputs from each group. Different groups can focus on extracting distinct types of low-level features that differentiate flames and smoke—for example, some groups capture brightness and sharp edges typical of flames, while others learn the more diffuse texture and lower contrast patterns characteristic of smoke. As a result, the model effectively reduces feature confusion between flame and smoke targets. This design allows for more efficient feature extraction without increasing the number of model parameters, thereby improving overall model performance.

In summary, in the baseline model Oriented R-CNN, the ResNeXt network was employed to replace the original ResNet backbone. This not only reduced the computational complexity of the model but also enhanced its adaptability to the complex variations in flame and smoke targets, ultimately improving detection performance.

### 2.5. Introducing the CARAFE Operator to Construct the FPN-CARAFE Layer Structure

In wildfire detection tasks, images typically contain objects of varying scales, particularly in the early stages of a fire, when small-scale flames and smoke targets are prevalent. Traditional feature up-sampling operators in the feature pyramid network (FPN) layer, such as nearest neighbor interpolation and bilinear interpolation [32], are commonly used. While these operators can handle multi-scale features, they often result in the loss of fine-grained details during up-sampling. This is especially problematic when detecting small and blurred targets.

**Figure 6:**
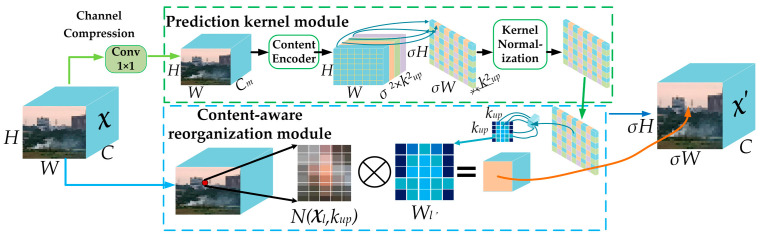
Work principles of CARAFE.

To address the issue of decreased detection accuracy in traditional FPN layers due to the loss of feature information during up-sampling, especially when dealing with small targets, this paper adopted the CARAFE (Content-Aware ReAssembly of FEatures) perceptual up-sampling operator. Unlike traditional interpolation methods, CARAFE preserves details more effectively by using content-aware weight allocation during the up-sampling process. Specifically, CARAFE determines how to fill in new pixel values based on the feature content at each position, rather than relying solely on neighborhood information. This approach better preserves the fine-grained details of the feature map during up-sampling, avoids the “distortion” issues commonly seen with traditional up-sampling methods, and is more suitable for small target detection tasks. The working principle of CARAFE is illustrated in Figure 6.

The workflow of the CARAFE operator consists of two main stages. In the first stage, the feature map χ, which contains the target position information, is channel-compressed by the channel compressor. This compressed feature is passed through a lightweight content encoder that predicts a unique reassembly kernel (up-sampling kernel) for each spatial location. These kernels represent weights over the local neighborhood pixels around the target up-sampled position. The kernel normalizer then applies softmax normalization to these weights, forming a probability distribution that dynamically emphasizes important details—such as edges and small targets like smoke and flames—while suppressing less relevant areas. In the second stage, based on the up-sampling rate σ, the predicted reorganization kernel is used to reorganize the original feature map, producing a new feature map $\chi^{\prime }$ of size C × σH × σW, thus achieving up-sampling.

Specifically, we adopted the CARAFE configuration of up-sampling kernel size 5, encoder kernel size 3, encoder dilation 1, and group number 1. These settings followed the recommendations of the paper in [26] and were empirically validated in our experiments as a good trade-off between accuracy and computational efficiency.

In summary, the CARAFE operator was introduced as the up-sampling method in the FPN layer of the model’s feature pyramid network to construct the FPN-CARAFE layer structure. Through its content-aware up-sampling mechanism, CARAFE more effectively preserved the fine-grained feature information of small targets, enhancing the model’s detection accuracy for small target flames and smoke.

## 3. Experimental Results and Analysis

### 3.1. Dataset Creation

The dataset used in this study was a self-constructed wildfire dataset. The original images were primarily captured on-site by an affiliated maintenance company, supplemented by a small number of publicly available images from the internet, resulting in a total of 1894 wildfire images. However, due to the limited data sources and insufficient quantity, the dataset exhibited significant category imbalance, which would likely lead to poor model performance if used directly for training.

**Figure 7:**
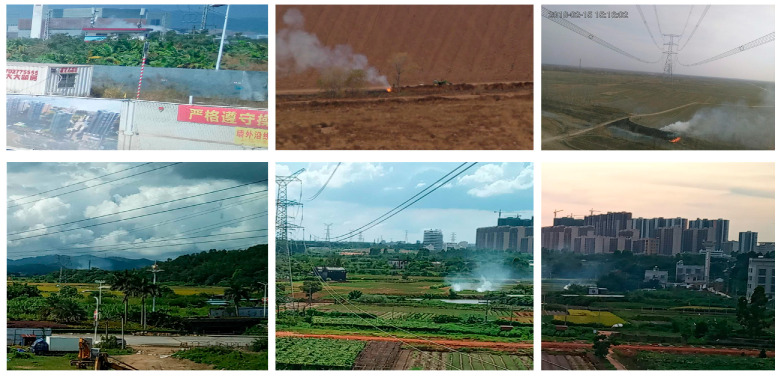
Transmission corridor wildfire dataset example.

To address this issue, data augmentation techniques such as cropping, rotation, and resizing were employed to expand the dataset and improve model robustness. Specifically, a sliding window cropping strategy with a window size of 1024 × 1024 and 50% overlap was used. Only image patches containing valid rotated bounding boxes were retained, and the angle information was preserved. Additionally, some image patches were randomly augmented using rotation within ±15°, resizing, and horizontal flipping. After augmentation, the final dataset contained 22,999 images, which were used for the experiments in this paper. Example images from the dataset are shown in Figure 7.

Labeling software was used to generate the corresponding XML annotation file for each image, in which the wildfire targets were categorized into two classes: “shanhuo” (flame) and “yanwu” (smoke). The dataset was then randomly divided into training, validation, and test sets in a ratio of 8:1:1, comprising 18,399, 2300, and 2300 images, respectively. To ensure reliable evaluation of the model’s robustness, the training, validation, and test sets were strictly separated with no image overlap. For consistency with the original annotation format, these Pinyin labels were retained in the model outputs and evaluation results. The composition of the dataset is shown in Table 1.

### 3.2. Experimental Platform and Training Parameters

The network model for detecting wildfires in power transmission corridors was trained on a high-performance computing platform. The detailed configuration of the experimental training environment is presented in Table 2.

The training hyperparameters for the model in this study were configured as follows: The total number of training epochs was set to 50, with a batch size of 4. The optimizer used was stochastic gradient descent (SGD), with an initial learning rate of 0.005, a momentum of 0.9, and a weight decay coefficient of 0.0001. The learning rate adjustment followed the step schedule, incorporating a linear warm-up strategy. Specifically, the warm-up lasted for 500 iterations, with a warm-up ratio of 1/3. Learning rate decay was applied at the 46th and 49th epochs. Gradient clipping was employed using the maximum norm method, with the maximum norm set to 35 and the norm type defined as the L2 norm.

### 3.3. Evaluation Indicators and Model Training

To objectively evaluate the performance of the improved model proposed in this study for wildfire target detection in power transmission lines, several evaluation metrics were adopted, including precision (P), recall (R), average precision (AP), mean average precision (mAP), and the number of network parameters. These metrics served as the basis for performance assessment.

The average precision (*AP*) measured the area under the precision–recall (*P*-*R*) curve for a single class and reflected the trade-off between precision and recall. It was calculated as the integral of precision with respect to recall. The mean average precision (*mAP*) was the mean value of APs across all object categories.

The calculation formulas for the evaluation indicators are as follows:

(15) $$P=\frac{N_{TP}}{N_{TP}+N_{FP}}\times 100\%$$

(16) $$R=\frac{N_{TP}}{N_{TP}+N_{FN}}\times 100\%$$

(17) $$AP=\int_{0}^{1}p(r)dr$$

(18) $$mAP=\frac{1}{m}\sum_{i=1}^{m}P_{Ai}$$

Among them, $N_{TP}$ is the number of correct detections predicted as positive samples that are actually positive samples; $N_{FP}$ is the number of false detections predicted as positive samples that are actually negative samples; $N_{FN}$ is the number of missed detections predicted as negative samples that are actually positive samples; $N_{TP}$ + $N_{FP}$ is the total number of samples judged as positive; $N_{TP}$ + $N_{FN}$ is the total number of positive samples; and ${AP}_{i}$ is the average accuracy of the ith category target. The calculation method was to draw the P-R curve of the ith category target—*p*(r) is the value of each point on the P-R curve—and integrate the curve to obtain the average detection accuracy AP of Formula (11). m in Formula (12) is the number of categories of targets to be detected.

### 3.4. Ablation Experiment

To verify the effectiveness of the three optimization modules proposed in this study and to evaluate their impact on the wildfire detection task in power transmission corridors, ablation experiments were conducted using the custom-built dataset. Starting from the baseline model Oriented R-CNN, the multi-feature center metric loss (MCM-Loss) module, the group convolutional network ResNeXt, and the FPN-CARAFE layer structure with perceptual up-sampling were added incrementally. In total, four experimental configurations were tested by progressively integrating the optimization components. The detection performance of each model variant was evaluated, and the results are presented in Table 3.

As shown in Table 3, compared with the baseline model Oriented R-CNN, the improvements proposed in this paper enhanced the model’s recognition accuracy to varying degrees. In Experiment 1, after introducing the multi-feature center metric loss (MCM-Loss) module, the detection accuracy of the model increased by 1.7%. This module enhanced the model’s ability to distinguish smoke features through a metric learning mechanism, resulting in a 2.5% improvement in the recognition accuracy of smoke targets, effectively reducing false detections and missed detections and improving the overall detection performance.

Experiment 2 built upon Experiment 1 by replacing the original ResNet backbone with the group convolutional ResNeXt network. With nearly no increase in model parameters, the detection accuracy improved by 0.6%. This demonstrated that the group convolution structure of ResNeXt enabled the model to capture features of different dimensions—such as shape, color, and texture—from various channel domains, thereby enhancing the detection capability for flame and smoke targets with diverse morphologies.

In Experiment 3, the FPN-CARAFE layer structure incorporating the content-aware up-sampling operator CARAFE was introduced. CARAFE adaptively up-sampled features based on local content, allowing the model to better retain fine-grained information of small-scale flames and smoke in the early stages of a wildfire. This enhancement led to a 4.1% improvement in overall detection accuracy, with the average precision (AP) of flame targets increasing by 8.1%, highlighting the model’s stronger capability in small object detection.

**Figure 8:**
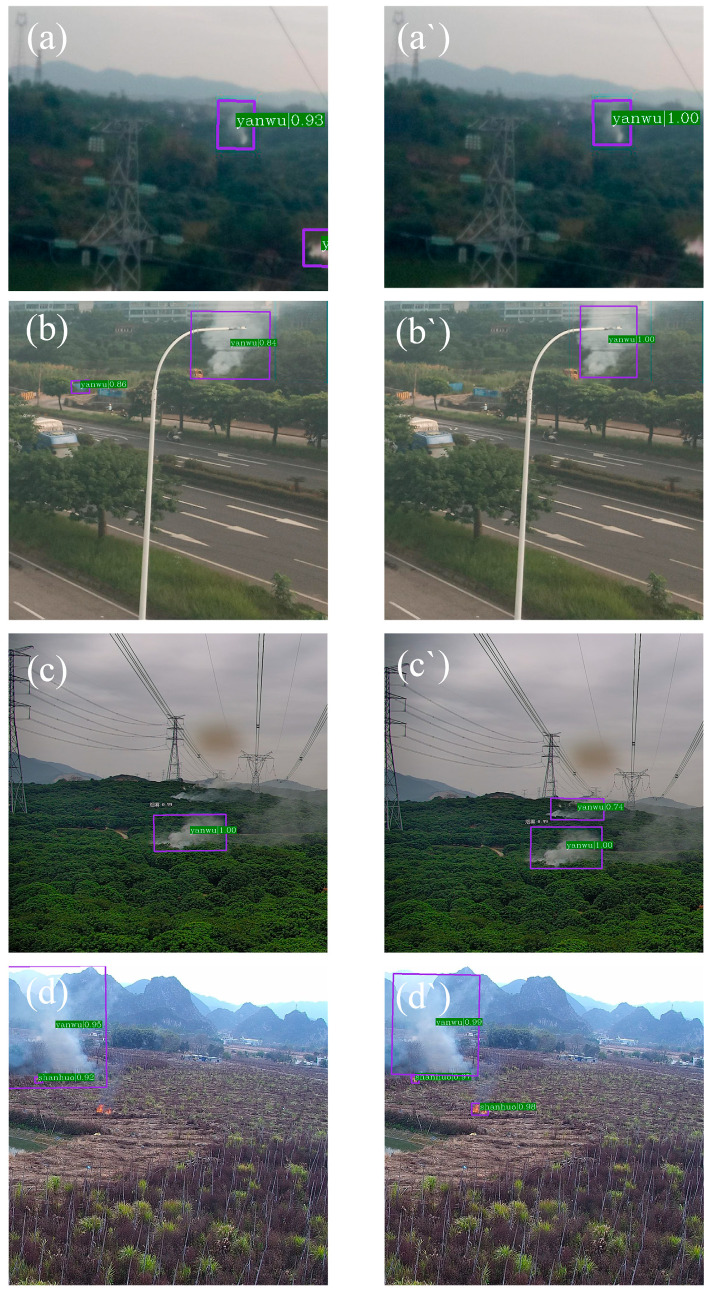
Comparison of detection effects between the baseline model Oriented R-CNN (subfigures **a**–**d**) and the improved model in this paper (subfigures **a′**–**d′**).

To more intuitively demonstrate the detection performance of the improved model proposed in this paper, several test set samples were selected to compare the baseline Oriented R-CNN model with the improved version in real detection tasks, as shown in Figure 8. Subfigures (a–d) present the detection results of the baseline Oriented R-CNN model, while (a′–d′) show the detection results of the improved model.

By comparing (a) and (b) with (a′) and (b′) in Figure 8, it can be observed that the baseline model tended to misidentify background areas—such as lake reflections—as smoke targets. After introducing the multi-feature center metric loss (MCM-Loss) module and replacing the backbone with the group convolutional ResNeXt network, the improved model effectively reduced such false detections and significantly enhanced its ability to distinguish between easily confused samples.

Furthermore, from the comparison of (c) and (d) with (c′) and (d′), it can be seen that the baseline model suffered from missed detections of small-scale flame and smoke targets. By integrating the content-aware up-sampling operator CARAFE into the FPN layer, the improved model significantly boosted its capability in detecting small objects, enabling it to successfully identify most low-resolution targets in the images.

In conclusion, these experimental results further validated the effectiveness of the three proposed enhancements—MCM-Loss, ResNeXt, and FPN-CARAFE—within the Oriented R-CNN framework, demonstrating notable improvements in detection accuracy for wildfire recognition tasks in power transmission corridors.

### 3.5. Comparative Experiment

To evaluate the performance of the proposed model more comprehensively, in addition to comparing it with the baseline Oriented R-CNN model, we also selected several current mainstream detection algorithms for comparison. These included the mainstream single-stage detector YOLOv8-l and several high-performing rotated object detection algorithms in recent years, such as ReDet, RoI Transformer, Rotated Faster R-CNN, and Gliding Vertex. Moreover, to provide a more comprehensive comparison with other rotated object detection models, we additionally included the combination of the recent Swin Transformer backbone with the RoI Transformer detection head (denoted as Swin-T RoI Transformer) as a new comparison model. All experiments were conducted on the same self-constructed dataset. The detection results are presented in Table 4 to facilitate a thorough evaluation of the proposed model’s performance and advantages in real-world wildfire detection scenarios for power transmission corridors.

**Figure 9:**
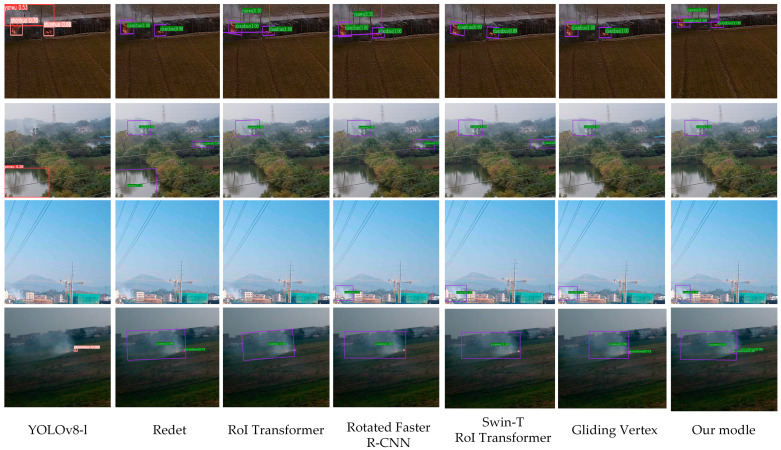
Visual comparison of different algorithms for wildfire smoke detection.

The experimental results demonstrated that the proposed model achieved the best performance in the wildfire detection task, with both precision and recall reaching the highest levels. Compared to other algorithms, the overall detection accuracy of the proposed model surpassed YOLOv8, ReDet, RoI Transformer, Rotated Faster R-CNN, Swin-T RoI Transformer, and Gliding Vertex by 14.4%, 9.2%, 9.1%, 5.7%, 5.6%, and 5.2%, respectively. The visual detection results of several test samples are illustrated in Figure 9.

Based on the results of the comparative experiments, the performance of various network models in wildfire detection tasks was analyzed and discussed. YOLOv8, as a typical single-stage detection algorithm, lacks fine-grained feature alignment mechanisms such as RoIAlign, making it difficult to accurately identify small-scale, blurred, or translucent flames and smoke targets under complex backgrounds. This results in a certain disadvantage in its overall accuracy in wildfire detection tasks. In contrast, two-stage detection models such as ReDet, RoI Transformer, Rotated Faster R-CNN, Swin-T RoI Transformer, and Gliding Vertex have stronger regional positioning and feature extraction capabilities. Specifically, after introducing the rotating anchor mechanism, these models can more effectively adapt to the detection needs of non-directional smoke and flame targets. However, these models still face limitations when dealing with the task of wildfire detection in power transmission corridors under complex backgrounds. These limitations are reflected in false detections of similar areas, such as reflective water surfaces and the omission of some real targets, which leads to lower precision, recall, and mAP scores, compared to the improved model proposed in this paper.

The improved Oriented R-CNN model introduced in this paper incorporated the multi-feature center metric loss (MCM-Loss) module, the group convolutional network ResNeXt, and the FPN-CARAFE layer structure with a perceptual up-sampling mechanism. These enhancements improved the model’s ability to distinguish similar samples and enhance feature learning for small targets, thereby improving the model’s recognition of flames and smoke. Comprehensive experimental results showed that, compared with other common algorithms, the model proposed in this paper achieved higher accuracy and recall rates in wildfire detection tasks. It significantly reduced the risk of missed detection and false detection and could accurately recognize and locate multi-type and multi-scale flame and smoke targets in complex environments.

## 4. Conclusions

Aiming to address the challenges in power transmission line wildfire detection, such as the presence of smoke-similar samples, diverse target morphologies, and the difficulty of detecting small target flames and smoke, this paper proposed a wildfire detection method for power transmission corridors based on an improved Oriented R-CNN with metric learning. The key conclusions were the following.

This paper constructed a high-quality dataset that included flame and smoke targets around transmission lines and proposed a wildfire detection method based on the Oriented R-CNN model. This method integrated the multi-feature center metric loss module (MCM-Loss), group convolution ResNeXt, and a fusion-aware up-sampling method. Experimental results demonstrated that the proposed model outperformed other widely used detection algorithms in terms of key indicators, such as recall and mAP.

The proposed MCM-Loss module improved the model’s ability to distinguish smoke from visually similar backgrounds. The ResNeXt backbone enhanced feature representation without significantly increasing the model complexity. The FPN-CARAFE structure further boosted small target detection by refining multi-scale feature fusion.

Moreover, the overall model maintained a relatively small parameter size, suggesting its potential for deployment on edge devices in transmission line scenarios.

However, there are still some limitations in the current study. First, the detection accuracy of smoke instances in highly cluttered backgrounds remains to be further improved. Second, although the model is relatively lightweight, its real-time inference speed on embedded devices has not been fully evaluated.

In future works, we will focus on lightweight network design (e.g., model pruning and structure simplification) and real-time performance optimization, aiming to achieve accurate and efficient wildfire detection in resource-constrained environments.

## Figures and Tables

**Table 1:** Composition of the wildfire dataset for transmission corridors.

Dataset	Flame (Shanhuo) Number of Instances	Smoke (Yanwu) Number of Instances	Dataset Partitioning
Transmission Corridor Wildfire Dataset	1661	2182	8:1:1

**Table 2:** Experimental platform parameters.

Operating System	CPU	GPU	Memory
Ubuntu 20.04	Dual-core Intel Xeon E5-2640v4 2.4 GHz (Intel Corporation, Santa Clara, CA, USA)	4 Nvidia Tesla V100 16 GB NVLink (NVIDIA Corporation, Santa Clara, CA, USA)	128 GB ECC DDR4

**Table 3:** Ablation experiment results.

Model	MCM-Loss	ResneXt	FPN-CARAFE	P/%	R/%	AP/%		mAP/%	Parameters/Million
						Flame (Shanhuo)	Smoke (Yanwu)		
Baseline Model				86.7	85.8	80.8	87.2	84.0	41.13
Exp 1	√			88.4	88.2	81.7	89.7	85.7	42.79
Exp 2	√	√		89.0	89.1	82.5	90.0	86.3	42.79
Exp 3	√	√	√	95.8	90.5	90.6	90.2	90.4	48.40

Exp 1, Exp 2, and Exp 3 refer to Experiment 1, Experiment 2, and Experiment 3, respectively.

**Table 4:** Comparative experiment results.

Model Name	P/%	R/%	AP/%		mAP/%	Parameters/Million
			Flame (Shanhuo)	Smoke (Yanwu)		
YOLOv8-l	79.9	76.4	74.5	77.5	76.0	43.61
Redet	87.8	82.3	81.8	80.5	81.2	33.37
RoI Transformer	86.0	85.2	81.8	80.8	81.3	55.12
Rotated Faster R-CNN	86.5	86.3	81.9	87.4	84.7	41.14
Swin-T RoI Transformer	86.2	87.1	82.8	86.7	84.8	58.75
Gliding Vertex	89.7	87.9	83.2	87.2	85.2	41.16
Our model	95.8	90.5	90.6	90.2	90.4	48.40

## Data Availability

The datasets presented in this article are not publicly available because they are self-built and contain sensitive information. Requests to access the datasets should be directed to (email: 2024202070023@whu.edu.cn).
